# High Precision UTDR Measurements by Sonic Velocity Compensation with Reference Transducer

**DOI:** 10.3390/s140711682

**Published:** 2014-07-02

**Authors:** Sam Stade, Mari Kallioinen, Mika Mänttäri, Tuure Tuuva

**Affiliations:** 1 Laboratory of Separation Technology, LUT Chemtech, Lappeenranta University of Technology, P.O. Box 20, Lappeenranta FI-53851, Finland; E-Mails: stade@lut.fi (S.S.); mika.manttari@lut.fi (M.M.); 2 Laboratory of Physics, Department of Mathematics and Physics, Lappeenranta University of Technology, P.O. Box 20, Lappeenranta FIN-53851, Finland; E-Mail: tuure.tuuva@lut.fi

**Keywords:** ultrasonic time-domain reflectometry, time-of-flight measurement, reference transducer, environmental compensation, flat-sheet membrane module

## Abstract

An ultrasonic sensor design with sonic velocity compensation is developed to improve the accuracy of distance measurement in membrane modules. High accuracy real-time distance measurements are needed in membrane fouling and compaction studies. The benefits of the sonic velocity compensation with a reference transducer are compared to the sonic velocity calculated with the measured temperature and pressure using the model by Belogol'skii, Sekoyan *et al.* In the experiments the temperature was changed from 25 to 60 °C at pressures of 0.1, 0.3 and 0.5 MPa. The set measurement distance was 17.8 mm. Distance measurements with sonic velocity compensation were over ten times more accurate than the ones calculated based on the model. Using the reference transducer measured sonic velocity, the standard deviations for the distance measurements varied from 0.6 to 2.0 μm, while using the calculated sonic velocity the standard deviations were 21–39 μm. In industrial liquors, not only the temperature and the pressure, which were studied in this paper, but also the properties of the filtered solution, such as solute concentration, density, viscosity, *etc.*, may vary greatly, leading to inaccuracy in the use of the Belogol'skii, Sekoyan *et al.* model. Therefore, calibration of the sonic velocity with reference transducers is needed for accurate distance measurements.

## 1. Introduction

Ultrasonic time-domain reflectometry (UTDR) can be used to measure distances accurately on the micrometer scale. It is based on time-of-flight measurement. The UTDR technique is simple and it requires only a transducer, a pulser and an oscilloscope to perform experiments in a laboratory. For industrial applications, data collection with a multiplexer and data processing with software are required to achieve real-time monitoring. UTDR has been compared to other non-invasive measuring techniques in the field of membrane technology and has been mentioned as one of the few methods which could be applied to commercial-scale modules [1].

UTDR has been successfully used in real-time monitoring of membrane compaction [2–5], fouling [6–9], cleaning [10,11] and membrane casting processes [12]. Compaction measurements have been done by measuring how much the time-of-flight from sensor to membrane increases. In fouling experiments, the membrane is usually first pre-compacted for stabilization before the fouling solution is added to the process. After that, the time-of-flight decreases when the fouling layer grows on top of the membrane. Usually the UTDR monitoring of membrane processes has been carried out with constant sonic velocity (C), which has a great impact on the measurement system accuracy, because C depends on the media and conditions in which the measurements are conducted. Typical factors affecting the C are temperature, pressure and changes in the process feed concentration or characteristics leading to changes in fluid compressibility or density. In some cases, the Belogol'skii, Sekoyan *et al.* model [13] or another similar model for C could be used to calculate the C in water to improve the accuracy of the UTDR measurements. However, the temperature and pressure of the media has to be measured from the same place as the UTDR measurement to achieve an accurate estimation for the C. In practice this is very difficult to do non-invasively.

This study demonstrates how the accuracy of the UTDR monitoring system can be improved by measuring C non-invasively with an additional reference transducer which measures a fixed distance in the flat-sheet membrane module. This measuring technique enables sonic velocity compensation and accurate UTDR measurements, which is especially important in real-time monitoring in fundamental studies of membrane compaction or swelling, fouling mechanisms or membrane formation, where C may vary due to changes in process conditions.

## 2. Ultrasonic Time-Domain Reflectometry

UTDR is based on high frequency sound waves, 10 MHz in this case, which travel through the media. When the high frequency ultrasonic wave encounters other media with a different density, some of the energy of the wave is reflected back. The reflected wave travels back to the sensor and it can be seen in the oscilloscope as an “echo”. Time-of-flight can be measured with an oscilloscope and distance can be calculated by Equation (1):

(1) $$\Delta s=\frac{1}{2}C\Delta t$$

where *Δs* is the distance from the transducer to the target, *C* is the sonic velocity in the media, and *Δt* is the measured time [6]. *C* depends on the media and the process conditions prevailing in the monitored process. The estimation of *C* to be constant causes inaccuracy in the measurements.

**Figure 1.**
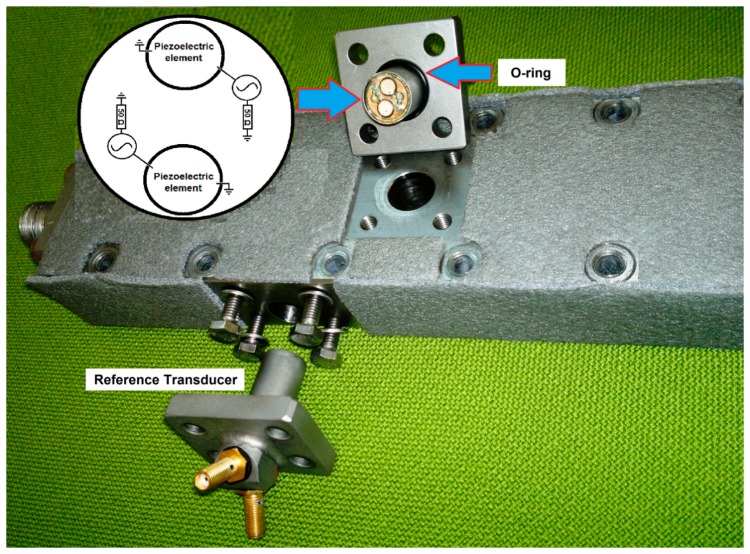
Two transducers with double piezoelectric elements are integrated inside the upper part of the membrane filtration module used in the study. Flow channel is inside the upper part of the module.

In the setup used in this study, *C* was measured with a reference transducer when the time-of-flight distance was known. The reference transducer was sideways in the filtration channel and it measured the distance between the walls of the flow channel, e.g., the channel width (17.8 mm). The main transducer measured the time-of-flight to the aluminium foil surface. Instead of polymeric membranes, aluminium foil was used to avoid the compaction of the target matrix, which would have been causing error in the determination of the accuracy of the UTDR system. Fifty (50) Ω termination resistors in the transducers were used to prevent oscillations due to cabling. The transducers were integrated in the membrane module (Figure 1) and they were immersed in the fluid inside the module. Both transducers were monitored simultaneously with an oscilloscope and the time-of-flight was measured from the first rising peak of the reflected echo. The membrane module was a cross-flow type, which means that the fluid was flowing along the channel over the membrane. Thus, fluid flowed tangentially to the measurement direction of the transducers. The flow channel was 17.8 mm high and wide and 310 mm long. Flow velocity and pressure were controlled with a pump before and a valve after the module. The temperature was measured from the concentrate stream after the module. The temperature was expected to be close to that inside the module, when the flow speed was 1.4 L/min. Pipes and module were also thermally insulated with foam plastic. Schematic drawing of the module and setup has been earlier explained in details by Stade *et al.* [5].

The measurements were performed at 0.1, 0.3 and 0.5 MPa. The temperature was increased from 25 °C to 60 °C, during which the distances were continuously monitored with UTDR. The measurements were carried out with reverse osmosis purified water (conductivity ∼1 μS/cm). Water was circulated through the module back to the feed vessel with a pump.

To explore the improvement of the reference transducer, the distance was obtained in two different ways, *i.e.*, using the reference transducer and calculating *C* with the model by Belogol'skii, Sekoyan *et al.* [13].

The distance with sonic velocity compensation was determined in two steps. First, *C* was calculated from Equation (1) using the constant filtration channel width as the distance *Δs* and the reference transducer measured time as *Δt*. Second, the calculated *C* was used with Equation (1) again, but *Δt* was now the time measured with the main transducer and the result was now the distance from the main transducer to the aluminum foil.

The results were compared with the results obtained when the *C* for the distance measurement was calculated with the model of Belogol'skii, Sekoyan *et al.* using the measured temperature and pressure (Equations (2)–(6), Table 1). Calculated *C* from the model was used then similarly than the reference transducer determined *C* in the Equation (1).

(2) $$C(T,P)=C(T,0)+M_{1}(T)(P-0.101325)+M_{2}(T){\left(P-0.101325\right)}^{2}+M_{3}(T){\left(P-0.101325\right)}^{3}$$

where *C*(*T*, 0), *M*_1_,*T*, *M*_2_,*T* and *M*_3_*T* are:

(3) $$C\left(T,0\right)=a_{00}+a_{10}T+a_{20}T^{2}+a_{30}T^{3}+a_{40}T^{4}+a_{50}T^{5}$$

(4) $$M_{1}\left(T\right)=a_{01}+a_{11}T+a_{21}T^{2}+a_{31}T^{3}$$

(5) $$M_{2}\left(T\right)=a_{02}+a_{12}T+a_{22}T^{2}+a_{32}T^{3}$$

(6) $$M_{3}(T)=a_{03}+a_{13}T+a_{23}T^{2}+a_{33}T^{3}$$

Temperatures are in degrees Celsius and pressures in megapascals.

## 3. Results and Discussion

**Figure 2.**
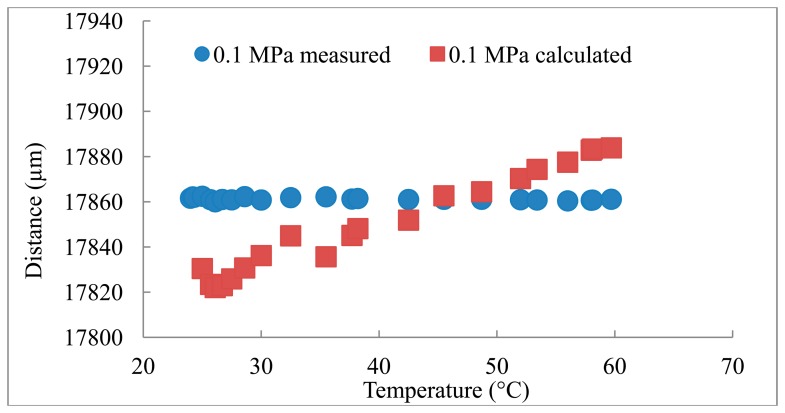
Experiment in 0.1 MPa pressure. σ_measured_ = 0.6 μm, σ_B&S_ = 20.8 μm.

**Figure 3.**
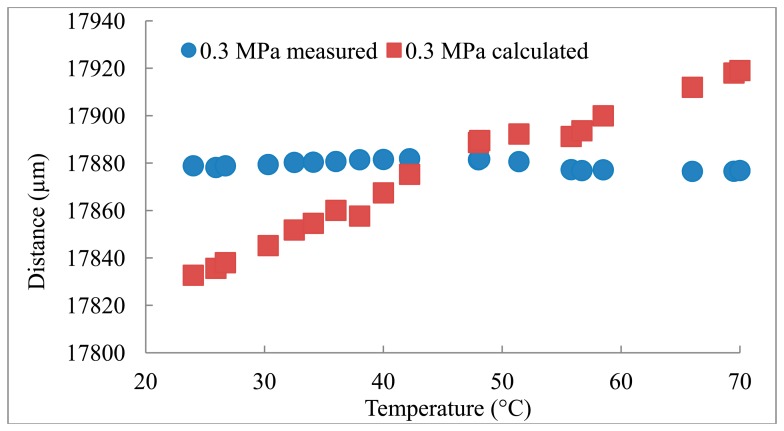
Experiment in 0.3 MPa pressure. σ_measured_ = 2.0 μm, σ_B&S_ = 28.1 μm.

**Figure 4.**
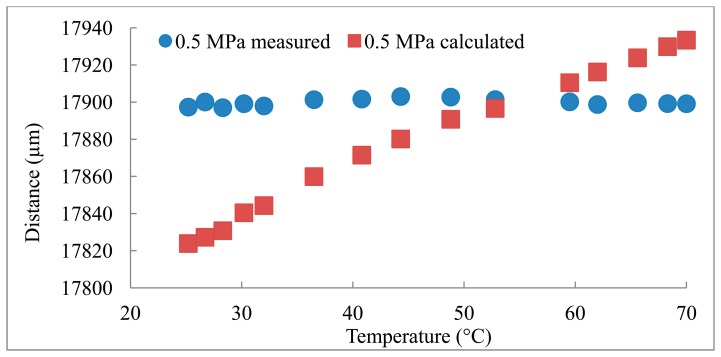
Experiment in 0.5 MPa pressure. σ_measured_ = 1.8 μm, σ_B&S_ = 39.2 μm.

**Figure 5.**
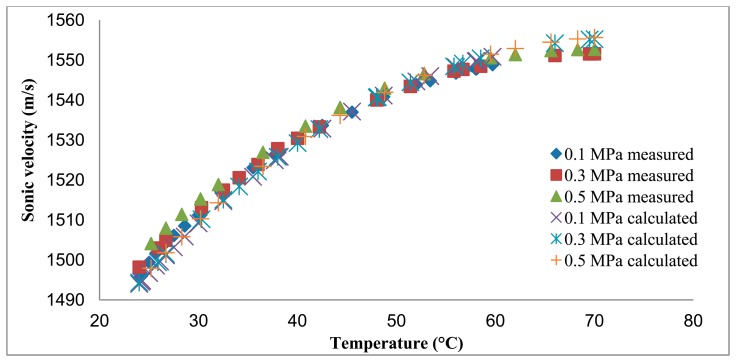
Ultrasonic reference transducer measured sonic velocities and calculated from the Belogol'skii, Sekoyan *et al.* model using measured temperature and pressure.

The distances measured using the reference transducer to measure *C* are compared to the distances calculated based on the *C* obtained from the Belogol'skii, Sekoyan *et al.* model in Figures 2–4. It can be seen that when the reference transducer is used to achieve the correct *C*, the measured distance varies significantly less than when the *C* based on the model of Belogol'skii, Sekoyan *et al.* is used. The difference between the calculated (Belogol'skii, Sekoyan *et al.*) and measured (reference transducer) *C* is small—only 0.2%—but it results in notable differences in distance measurements at the micrometer level (Figure 5). The small differences in Figure 5 originate from inaccuracy in the temperature measurements, the accuracy of the temperature meter and its calibration. In addition, the water used in these measurements was not exactly of the same purity as that used by Belogol'skii, Sekoyan *et al.* In industrial scale operations, the feed stream concentration and conditions may vary by more than this experiment, which favors the use of the reference transducer.

The distance measured in the experiment was height of the flow channel, 17.8 mm, which was confirmed with micrometer measurements. However, the actual distance between the main transducer and the aluminum foil is a little longer (∼0.1 mm) as the module is designed for polymeric membranes which are thicker than the aluminum foil. During the experiment the distance is constant and this was supported by the measured UTDR data (Figures 2–4). The standard deviations (σ) of the distance measurements were 0.6, 2.0 and 1.8 μm when the measurements were carried out with the reference transducer at pressures of 0.1, 0.3 and 0.5 MPa. The corresponding values using the *C* calculated from the Belogol'skii, Sekoyan *et al.* model were 20.8, 28.1 and 39.2 μm. The small difference in distances measured at different pressures originates from the deformation of the aluminum foil on the supporting spacer at the lower part of the filtration module. The spacer is a porous metal plate and at increased pressure the aluminum foil deforms slightly into the pores. This does not affect in the sonic velocity compensation which is performed with the reference transducer.

Temperature change influences the *C* more than pressure change (Figure 5). The reason for this is that water density depends on the temperature, but water is an almost incompressible fluid and pressure has thus only a small effect on the *C* value. The reference transducer used in this study to measure *C* can also be calibrated to measure temperatures when the other parameters affecting the *C* remain constant.

As can be seen from the results, the UTDR tool equipped with the reference transducer provides remarkably greater accuracy than the UTDR tools without the possibility to calculate accurate *C* values in the prevailing conditions. Therefore, the UTDR tool equipped with the reference transducer provides new possibilities in the UTDR measurements used to monitor membranes or their fouling in the flat-sheet membrane modules. However, many membrane filtration plants are operating with spiral wound, tubular or hollow fiber modules. This type of UTDR technology with a reference transducer is challenging to use in these modules due to how the sonic velocity compensation could be established in them. One option would be to measure the *C* before and after the spiral wound or hollow fiber module and estimate the value inside the module with measured data. Sonic velocity compensation technology could also be used for flat-sheet membrane modules in by-pass flow with the spiral wound module. These modules have earlier been developed as fouling detectors for early warning systems [14–18]. Flat-sheet modules have been designed to have the same membrane, spacer and filtration conditions as spiral wound modules which have been flattened to achieve the properties of the spiral wound module to the extent possible. The accuracy of systems using UTDR to measure fouling layer thickness can be improved with the sonic velocity compensation technology.

## 4. Conclusions

This study examined the influence of certain *C* values on the accuracy of UTDR measurements. The distance values measured with the UTDR tool equipped with the reference transducer for *C* measurements were compared to the results achieved when the *C* calculated based on the model of Belogol'skii, Sekoyan *et al.* The use of the reference transducer for the calculation of *C* in the prevailing process conditions provided over ten times more accurate distance measurements than using the calculated *C* from the model. The σ improved from 20.8–39.2 μm to 0.6–2.0 μm. The results clearly demonstrate that the use of the reference transducer improves the possibilities to use UTDR monitoring when new information is gathered on phenomena such as membrane compaction, swelling and fouling, which occur on the micrometer scale. The possibility to decrease the influence of changes in process conditions on UTDR measurement results will also improve the applicability of UTDR monitoring in a wide variety of membrane processes.

## Figures and Tables

**Table 1.** Constants *a*_nn_ are listed below.

*a*_00_	1402.38744	*a*_31_	2.718246452 × 10^−6^
*a*_10_	5.03836171	*a*_02_	4.31532833 × 10^−3^
*a*_20_	−60.1172916	*a*_12_	−33.38590293
*a*_30_	3.34638117 × 10^−4^	*a*_22_	6.822485943 × 10^−6^
*a*_40_	−20.8259672	*a*_32_	−74.74551162
*a*_50_	3.16585020 × 10^−9^	*a*_03_	−23.52993525
*a*_01_	1.49043589	*a*_13_	1.481844713 × 10^−6^
*a*_11_	1.077850609 × 10^−2^	*a*_23_	−47.40994021
*a*_21_	−26.32794656	*a*_33_	3.939902307 × 10^−10^
